# S100P, a calcium-binding protein, is preferentially associated with the growth of polypoid tumors in colorectal cancer

**DOI:** 10.3892/ijmm.2015.2065

**Published:** 2015-01-12

**Authors:** JY-MING CHIANG, REPING TAN, JEN-YI WANG, JINN-SHIUM CHEN, YUN-SHIEN LEE, PAO-SHIU HSIEH, CHUNG RONG CHANGCHIEN, JIM-RAY CHEN

**Affiliations:** 1 Division of Colon and Rectal Surgery, Chang Gung Memorial Hospital at Linkou, Taiwan, R.O.C.; 2 College of Medicine, Chang Gung University, Taiwan, R.O.C.; 3 Genomic Medicine Research Core Laboratory, Chang Gung Memorial Hospital at Linkou, Taiwan, R.O.C.; 4 Department of Biotechnology, Ming Chuan University, Kwei-Shan, Tao-Yuan 333, Taiwan, R.O.C.; 5 Department of Pathology, Chang Gung Memorial Hospital at Keelung, Keelung 204, Taiwan, R.O.C.

**Keywords:** colorectal cancer, polypoid, ulcerative, cDNA micro-array, S100P

## Abstract

Colorectal cancer (CRC) is a genetically heterogeneous disease with distinct morphological patterns. It has been shown that polypoid and ulcerative CRC displays different genetic alterations. In the present study, we aimed to investigate genes with differential expression patterns between ulcerative and polypoid CRC. cDNA microarray analysis was performed to compare the gene expression profiles in samples of ulcerative and polypoid CRC with paired normal mucosa samples. Potential candidate genes were further validated using reverse transcription-quantitative polymerase chain reaction (RT-qPCR), western blot analysis and immunohistochemistry. The epigenetic regulation of gene expression was investigated using methylation-specific PCR (MSP). cDNA microarray analysis identified 11 upregulated and 14 downregulated genes which were differentially expressed in samples from both tumor types compared to the matched normal mucosa samples. Among these, S100P was the only upregulated gene preferentially associated with polypoid CRC (P=0.032). The samples of polypoid CRC displayed significantly higher S100P protein and mRNA expression levels than the samples of ulcerative CRC (P<0.05, respectively). Using semi-quantitative immunohistochemical analyses, S100P overexpression was found to be preferentially associated with polypoid CRC (24/30 vs. 14/40, P<0.001). The relative methylation level determined by MSP did not differ significantly between the samples of polypoid and ulcerative CRC (43.36 vs. 49.10%, P=0.168), indicating that promoter hypomethylation was not directly related to the upregulation of S100P mRNA. Our results demonstrate that the upregulation of S100P mRNA and protein expression is a predominant characteristic in polypoid CRC, whereas ulcerative CRC presents with a wide range of expression levels, indicating that S100P overexpression is not a key determinant in conferring invasion properties. The clinicopathological significance of S100P in CRC requires further investigation in well-controlled studies.

## Introduction

The molecular pathogenesis of colorectal cancer (CRC) is a complex multistep process involving multiple acquired genetic and epigenetic abnormalities. The molecular pathogenesis of CRC is among the most extensively studied of all human cancers. Based the histopathological and epidemiological data, the adenoma-adenocarcinoma sequence is generally regarded as the predominant pathogenetic pathway in colorectal carcinogenesis. A simplified genetic model of colorectal tumorigenesis involving a series of progressive molecular alterations was previously proposed based on the adenoma-adenocarcinoma sequence (1). Accumulative evidence indicates that CRC is a disease involving multiple pathways comprising distinct subgroups with particular clinicopathological and molecular characteristics (2). Morphologically, the growth pattern of a colorectal neoplasm is extremely diverse and has been endoscopically into divided 2 major categories: polypoid and non-polypoid. The latter can be further classified into flat, superficial, lateral spreading, depressed and ulcerative patterns (3). Polypoid and ulcerative lesions, the 2 most common types, represent upward (exophytic) and downward (endophytic) tumor growth, respectively, whereas flat or lateral spreading CRC demonstrates horizontal tumor expansion (3). Dome-type carcinoma, a rare variant of CRC characterized by well or moderately differentiated histology, expansive growth and a dense lymphoid stroma, has also been previously reported (4).

It has been demonstrated that these morphologic variants of colorectal adenoma and carcinoma harbour diverse genetic alterations and exhibit different clinicopathological characteristics (5–8). The presence of a precursor lesion, the adenoma, provides an ideal focus for molecular studies. Comparative genomic hybridization profiling has shown distinct chromosomal imbalances between polypoid and non-polypoid adenomas (9). Furthermore, non-polypoid colorectal adenomas have been shown to have loss of heterozygosity at chromosomes 3p (8), a high frequency of somatic mutations of the adenomatous polyposis coli (APC) and p53 genes (10), and a low KRAS mutation rate (11).

Different genetic abnormalities between polypoid and ulcerative CRC have also been reported. We, as well as others have previously reported that KRAS mutations are preferentially associated with polypoid CRC (12,13), whereas the nuclear expression of β-catenin expression occurs more frequently in ulcerative CRC (14,15). The aforementioned molecular genetic results are mainly based on the analysis of individual genes involved in the colorectal tumorigenesis pathway and cannot sufficiently explain the differences existing between polypoid and ulcerative CRC. cDNA microarray analysis has been successfully employed to study global gene expression profiles in a number of diseases (16). To the best of our knowledge, there are no expression data available to date on the differences between polypoid and ulcerative CRC. In the present study, we first employed cDNA microarray analysis to screen for genes differentially expressed between samples of normal mucosa and samples of polypoid or ulcerative CRC. Among all the potential dysregulated genes in both tumor types, S100P, a member of the S100 family of proteins containing 2 EF-hand calcium-binding motifs (17), was found to be upregulated and preferentially associated with polypoid CRC. To validate this finding, we further analyzed S100P mRNA and protein expression levels and the status of promoter methylation in an independent series of CRC and matched normal mucosa samples using different techniques.

## Materials and methods

### Ethical considerations

The present study was approved by the Institutional Review Board (IRB) of Chang Gung Memorial Hospital, Tao-Yuan, Taiwan.

### Tumor and normal tissue samples

CRC tissue samples were obtained under informed consent from patients who underwent colectomy at the Chang Gung Memorial Hospital at Linkou, Tao-Yuan, Taiwan. Fresh tumor tissue and matched normal mucosa samples at a distance of at least 10 cm from the tumor site were snap-frozen in liquid nitrogen and then stored at −80°C until use. Paired samples of normal mucosa and polypoid (n=8) and ulcerative (n=8) tumors were used in the cDNA microarray analysis. The classification of polypoid and ulcerative CRC was carried out as previously described (15). Namely, tumors with exophytic cauliflower-like appearances with or without a very shallow ulcer only and with a height exceeding half their diameter, were classified as polypoid. Tumors showing endophytic growth within depressed ulcers with or without very low elevated edges were classified as ulcerative.

### RNA isolation and cDNA microarray analysis

Total RNA was extracted from approximately 100 mg of individual tissue samples using TRIzol reagent (Invitrogen, Carlsbad, CA, USA) according to the manufacturer’s instructions. The integrity of the isolated RNA was assessed by denaturing agarose gel electrophoresis. A lab-on-a-chip device, RNA labchio, read on a Bioanalyzer 2100 (Agilent Technologies, Inc., Santa Clara, CA, USA) was used to evaluate the RNA quantity. The GMRCL Human 7K set, version 1 chips (InCyte Genomics, Palo Alto, CA, USA) containing 7,334 sequence-verified human genes were used in the present study. The fabrication of the slides, hybridization, washing and detection of signals were carried out at the Genomic Medicine Research Core Laboratory of Chang Gung Memorial Hospital at Linkou, as previously described (18). An indirect labeling of cDNA targets using the 3DNA Sunmicro EX Expression Array Detection kit (Genisphere, Hatfield, PA, USA) was used. Total RNA (10 *μ*g) from each experimental sample was reverse transcribed into target cDNA using an oligo-d(T) primer tagged with either Cy3- or Cy5-specific 3DNA-capture sequences. Following hybridization and washing, the slides were scanned using a confocal scanner ChipReader (Virtek Vision International, Waterloo, ON, Canada). The spot and background intensities were then acquired using GenePix Pro 4.1 software (Axon Instruments, Union City, CA, USA) and a two-step pre-analysis data management, including flooring and within-slide normalization, was performed using MATLAB 6.0 software (MathWorks, Natick, MA, USA). We averaged the log ratios of the duplicated spots on each slide in dye-swapping experiments. Genes with an average tumor-to-normal mucosa ratio of >2 or <0.5 were considered as significant candidate genes.

The data, including all raw microarray data have been deposited in the Gene Expression Omnibus (GEO) database with Accession no. GSE46905 (http://www.ncbi.nlm.nih.gov/geo/query/acc.cgi?acc=GSE46905).

### Reverse transcription-quantitative polymerase chain reaction (RT-qPCR)

Total RNA was extracted from an independent series of paired samples of polypoid (n=9) and ulcerative (n=9) tumors and normal mucosa using an RNeasy Mini kit (Qiagen, Valencia, CA, USA) and reverse transcribed into cDNA using SuperScript III first-strand synthesis (Invitrogen) and random hexamers. mRNA transcripts were measured using SYBR-Green-based quantitative PCR with Power SYBR-Green PCR Master Mix (Applied Biosystems, Foster City, CA, USA) and the ABI Prism 7700 sequencing detection system (Applied Biosystems). Primers were designed using Primer Express software version 2.0 (Applied Biosystems). The sequences of the primers used were as follows: human S100P forward, 5′-GACCTGGACGCCAATGGA-3′ and reverse, 5′-GTGATT GCAGCCACGACCAC-3′; human cytokeratin 20 (CK20) forward, 5′-AGTTCTGCAGCAACAGGTCACAG-3′ and reverse, 5′-CTTCCAGAAGGCGGCGGTAAGTAG-3′; and human glyceraldehyde-3-phosphate dehydrogenase (GAPDH) forward, 5′-GCTCAGACCAGCTCATACTTC ATG-3′ and reverse, 5′-GATAGGCATTGGTGCCTTCTG-3′. To normalize for variance in loaded cDNA, CK20 and GAPDH were amplified in separate reactions. All reactions were performed in triplicate, with water as the negative controls. Standard curves were constructed for each gene in each experiment to normalize the relative expression of S100P to the CK20 and GAPDH controls using the ΔCt method.

### Western blot analysis

Protein samples were isolated from an independent series of paired specimens of polypoid (n=10) and ulcerative (n=10) tumors and normal (non-cancerous) mucosa using PRO-PREP™ Protein Extraction Solution (iNtRON Biotechnology, Seoul, Korea). An equal amount of protein in each sample was quantified using the Bio-Rad protein assay kit (Bio-Rad Laboratories, Hercules, CA, USA). Cellular proteins were separated by 15% SDS-polyacrylamide gel electrophoresis and transferred onto nitrocellulose membranes. The membranes were blocked in 5% milk solution for 30 min at room temperature. The membranes were then incubated with rabbit monoclonal anti-S100P at a 1:1,000 dilution (clone EPR6143; Epitomics, Burlingame, CA, USA) at 4°C overnight, followed by incubation with IRDye polyclonal secondary antibodies (LI-COR Biosciences, Lincoln, NE, USA). Signals were detected using the ECL Detection system (Bio-Rad) as per the manufacturer’s instructions. Mouse monoclonal anti-cytokeratin AE1/AE3 antibody (cat. no. MAB3412) and mouse monoclonal anti-GAPDH antibody (cat. no. CB1001, both 1:1,000 dilution; Millipore, Billerica, MA, USA) were used as the controls for normalization. Densitometric analysis of the bands compared with the density of AE1/AE3 and GAPDH was performed using ImageJ software, as previously described (19).

### Methylation-specific PCR (MSP)

An independent series of paired formalin-fixed paraffin-embedded specimens of ulcerative (n=9) and polypoid (n=9) tumors and normal mucosa samples was used for MSP analysis. Using hematoxlyin and eosin-stained slides as a reference, 1-mm tissue core was punched directly from areas with a high (>90%) tumor content and areas of normal mucosa. DNA was isolated using the DNeasy kit (Qiagen). The DNA concentration was measured by spectrophotometry (serial no. 1402067; Biotech Instruments, Winooski, VT, USA). DNA (1 *μ*g) was subjected to sodium bisulfite treatment using the EZ DNA Methylation™ kit (Zymo Research, Irvine, CA, USA) according to the instructions provided by the manufacturer. The methylation status of the S100P gene was determined by MSP as described in a previous study (20). Primers were designed to detect the sequence differences between methylated and unmethylated DNA following bisulfite modification, and each primer pair contained at least 3 CpG sites to provide optimal specificity. Since the knowledge of the regulatory regions of the S100P gene in CRC is limited, we characterized the methylation status of CpG islands within a few hundred base pairs of the transcriptional start site, where CpG methylation has been implicated in transcriptional silencing (21). The sequences of the primers used were as follows: wild-type S100P forward, 5′-GCTGCCAGTGGGACATTTTCTCGG-3′ and reverse, 5′-CGCTGCCCGAATATCGGGAAAAGACG-3′; methylated forward, 5′-GTTGTTAGTGGGATATTTTGTCGGC-3′ and reverse, 5′-CGCTACCCGAATATCGAAAAAAAAC-3′ and unmethylated forward, 5′-GAGGTTGTTAGTGGGATATTT TTTTGGT-3′ and reverse, 5′-CTCACTACCCAAATATCAA AAAAAAACTC-3′. PCR was carried out at 95°C for 15 min as the first step, followed by 35 cycles of 95°C for 30 sec, 58°C for 1 min, and 72°C for 1 min. It has previously been demonstrated that S100P is aberrantly hypomethylated in primary pancreatic cancer (22); therefore, in this study, 3 cases of pancreatic adenocarcinoma and 1 case normal pancreas were also analyzed.

### Immunohistochemistry (IHC)

Formalin-fixed, paraffin-embedded sections of 35 adenomas and 70 paired samples of normal mucosa and adenocarcinomas (polypoid tumors, 30 and ulcerative tumors, 40) were used for IHC. The clinicopathological characteristics of the 70 patients included in IHC analysis are summarized in Table I. IHC was performed using a monoclonal rabbit antibody (anti-S100P; clone EPR6143; Epitomics, 1:800) as previously described (23). Briefly, 4 to 5-*μ*m-thick sections were deparaffinized in xylene, rehydrated in graded alcohol followed by antigen retrieval in 10 mmol/l citrate buffer pH 6.0 at 120°C for 10 min in a pressurized heating chamber (Biocare Medical, Concord, CA, USA). Immunostaining was carried out on a Dako Universal Autostainer Plus (Agilent Technologies) using DakoChemMate™EnVison™+ Detection kits (Agilent Technologies) in accordance with the manufacturer’s instructions. Signal detection was performed using 3,3′-diaminobenzidine as the chromagen. The slides were counterstained with hematoxylin, cleared in xylene and mounted with Permount. Placenta was used as a positive control. For the negative control, the primary antibody was replaced with phosphate-buffered saline.

The slides were independently examined by 2 observers (J.-M.C. and J.-R.C.) who were blinded to the clinicopathological data and to the initial results of the other observer. Staining intensity was evaluated semi-quantitatively by both the estimated average staining intensity of the nucleus and cytoplasm (graded as 0, no staining; 1, weak staining; 2, moderate staining; and 3, strong staining) and the proportion of positive cells (graded as 0, no positive cells; 1, 1–25% positive cells; 2, 26–50% positive cells; 3, 51–75% positive cells; and 4, >76% positive cells). The combination of both parameters results in a seven-step score. The overexpression of S100P in the tumors was defined by the score difference between the tumor and normal mucosa samples. Negative or weakly expressed cases had a score of 0–3; moderately expressed cases had a score of 4–5; and strongly overexpressed cases had a final score of 6 and 7. Images were acquired using an Eclipse 90i microscope (Nikon, Tokyo, Japan) equipped with a DP71 CCD camera image capture system (Olympus, Tokyo, Japan). The acquisition of the panoramic view of the tumors was achieved using an iScan Coreo slide scanner (Ventana Medical Systems, Inc., Tucson, AZ, USA).

### Statistical analysis

The expression levels of the upregulated and downregulated genes between the polypoid and ulcerative CRC samples were compared using the Student’s t-test. The χ^2^ test was performed to evaluate the correlations between clinicopathological parameters and the protein expression level of S100P. Both were performed using the Statistical Package for the Social Studies version 11 software (SPSS Inc., Chicago, IL, USA). Differences were considered significant at P<0.05. Results are expressed as the means ± standard deviation (SD), unless otherwise stated.

## Results

### Gene expression analysis by cDNA microarray

The results of cDNA microarray analysis revealed that the majority of the 7,334 genes detected had similar expression levels in both tumor types and matched normal mucosa samples. The array data were filtered to produce a list of 11 upregulated and 14 downregulated genes in both types of tumor compared with the matched mucosa samples (Table II). The majority of these genes have been previously reported, such as transforming growth factor-β, serine protease inhibitor, metallothionein, GRO3 oncogene regenerating islet-derived protein and butyr-ophilin. Among these 25 gene candidates, S100P was the only upregulated gene found to be preferentially involved in polypoid CRC (P=0.032); therefore, the S100P gene was investigated in more detail.

### Validation of S100P mRNA and protein expression in normal mucosa and CRC

**Figure 1:**
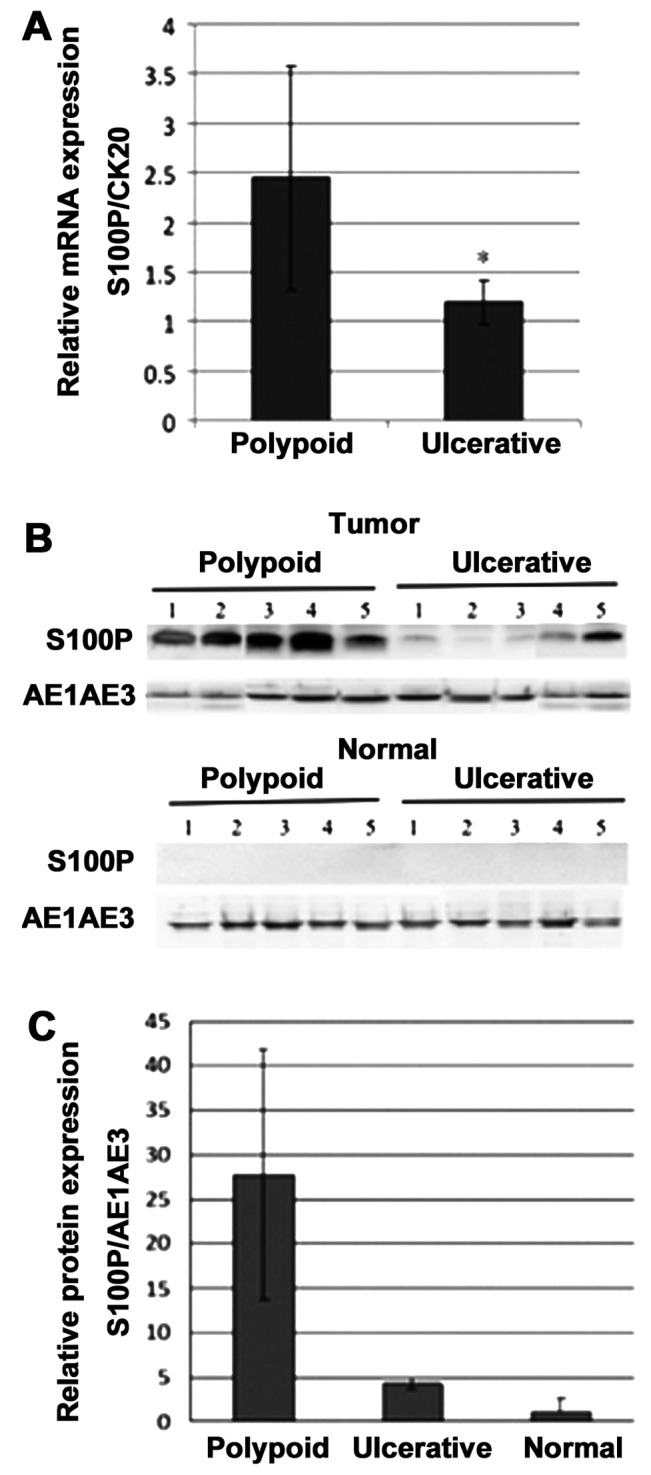
S100P mRNA and protein expression in polypoid and ulcerative colorectal cancer and matched normal mucosa samples. (A) The S100P mRNA relative expression level in polypoid tumors was significantly higher (−2.5-fold) than that in ulcerative tumors (P<0.05; Student’s t-test). (B) Representative western blot analysis showing S100P protein expression in polypoid and ulcerative tumor and paired normal colon mucosa samples. Pan-cytokeratin (AE1/AE3) was used as an internal control. (C) S100P protein relative expression level in polypoid (n=10) and ulcerative (n=10) tumor and paired normal colon mucosa samples. Data shown are the means ± SE from 3 independent experiments (P<0.05; Student’s t-test).

For verification, qPCR analysis revealed that S100P mRNA expression was detected in both types of tumor and their matched normal mucosa samples. The relative mRNA expression level (S100P/reference genes) in the polypoid and ulcerative tumors was 2.5- and 1.2-fold higher than that of the matched normal mucosa samples, respectively; these results were statistically significant (polypoid, P<0.01; ulcerative, P<0.05; Student’s t-test). Polypoid tumors showed a significantly higher expression level (2.5-fold increase) of S100P compared with the ulcerative tumors (P<0.05; Student’s t-test; Fig. 1A).

Using western blot analysis, S100P protein expression was barely detected in the normal mucosa samples (Fig. 1B). The relative protein expression level (S100P/internal control) in the polypoid and ulcerative CRC samples was 27.8- and 4.3-fold higher than that of the matched normal mucosa samples (Fig. 1C), respectively; these results were statistically significant (P<0.01; Student’s t-test). Polypoid tumors showed a significantly higher S100P expression level (6.5-fold increase) compared with the ulcerative tumors (P<0.05; Student’s t-test; Fig. 1C).

### Promoter methylation by MSP

**Figure 2:**
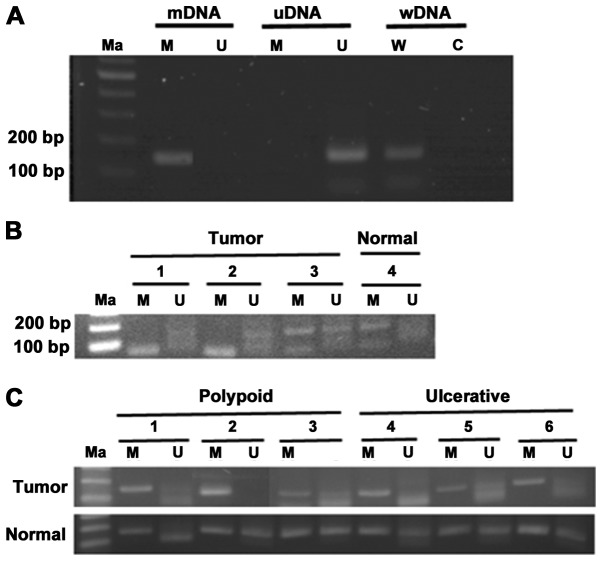
Representative methylation-specific PCR (MSP) of S100P promoter status. (A) MSP analysis of methylated control DNA (mDNA), unmethylated control DNA (uDNA) and untreated DNA (wDNA) using designed primers sets showing the specificity of primers. (B) MSP analysis of S100P in pancreatic cancer and paired normal tissue. (C) Representative results of MSP analysis of S100P in polypoid and ulcerative colon cancer and paired normal mucosa samples. The PCR products in lanes M and U indicate the presence of methylated and unmethylated templates, respectively. Ma, 100-bp ladder marker.

Previous data have indicated that a higher mRNA expression level of S100P is closely associated with theo hypomethylation of the S100P promoter (22). Therefore, in this study, we performed MSP to examine the methylation status of S100P in polypoid and ulcerative tumor samples. We first examined the specificity of our designed primers. DNA not treated with bisulfite was readily amplified with wild-type primers. Unmethylated and methylated control DNA was specifically amplified with unmethylated- and methylated-specific primers, respectively (Fig. 2A). We then examined the methylation status of 3 pancreatic adenocarcinomas and 1 normal pancreas. The hypermethylation of S100P was predominantly detected in the normal pancreas, whereas the 3 pancreatic adenocarcinomas showed variable methylation levels (Fig. 2B), similar to previous observations (22). The normal colon mucosa samples, which showed barely detectable S100P mRNA levels by qPCR, displayed predominant hypermethylation of S100P (Fig. 2C). Even though the polypoid CRC samples displayed significantly higher S100P mRNA expression levels compared with the ulcerative CRC samples, the methylation levels appeared to be similar in these 2 types of CRC (Fig. 2C). The relative methylation level (the relative proportion of methylated alleles against the total intensity of unmethylated and methylated alleles) did not differ significantly between the polypoid and ulcerative CRC samples (43.36 vs. 49.10%; P=0.168), indicating that promoter hypomethylation was directly related to the upregulation of S100P mRNA expression.

### Immunohistochemical characterization of S100P protein expression

**Figure 3:**
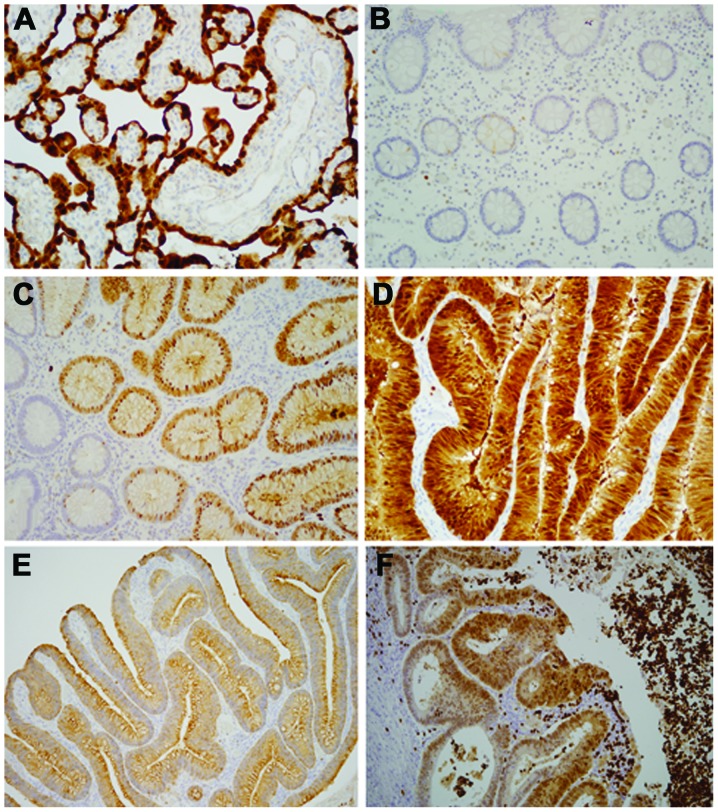
Immunohistochemical detection of S100P protein expression in placenta and colon tissue. (A) Trophoblasts lining the placental chorionic villi demonstrated uniform and strong staining (magnification, ×200). (B) Normal colonic mucosa showed negative staining (magnification, ×200). (C) Glands with low-grade dysplasia showed heterogeneous nuclear staining and weak cytoplasmic staining (magnification, ×200). (D) Strong nuclear and moderate cytoplasmic staining in glands with high-grade dysplasia (magnification, ×400). (E) Strong staining in the apical region of cytoplasm without nuclear expression (magnification, ×100). (F) Neutrophils forming abscess around invasive cancer displayed very strong immunostaining (magnification, ×200).

**Figure 4:**
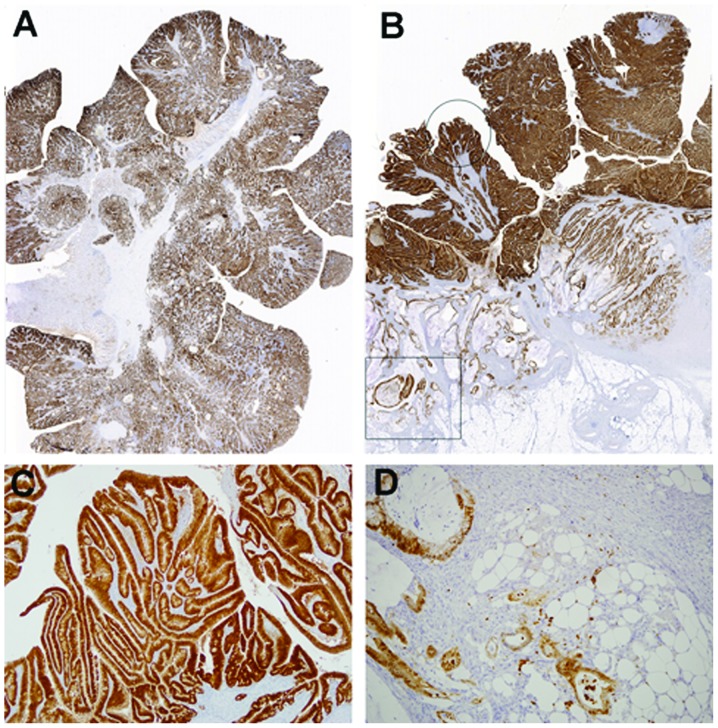
Representative S100P protein expression in polypoid tumor samples. (A) Diffuse, strong S100P immunostaining in a polypoid tumor with invasion to the submucosa (panoramic view). (B) Heterogeneous expression of S100P in a polypoid tumor with invasion to the adipose tissue (panoramic view). (C) Magnification of polypoid portion (circle) in (B) showing strong S100P immunoreactivity (magnification, ×100). (D) Magnification of invasive front (square) in (B) displaying diminished S100P expression (original magnification, ×400).

**Figure 5:**
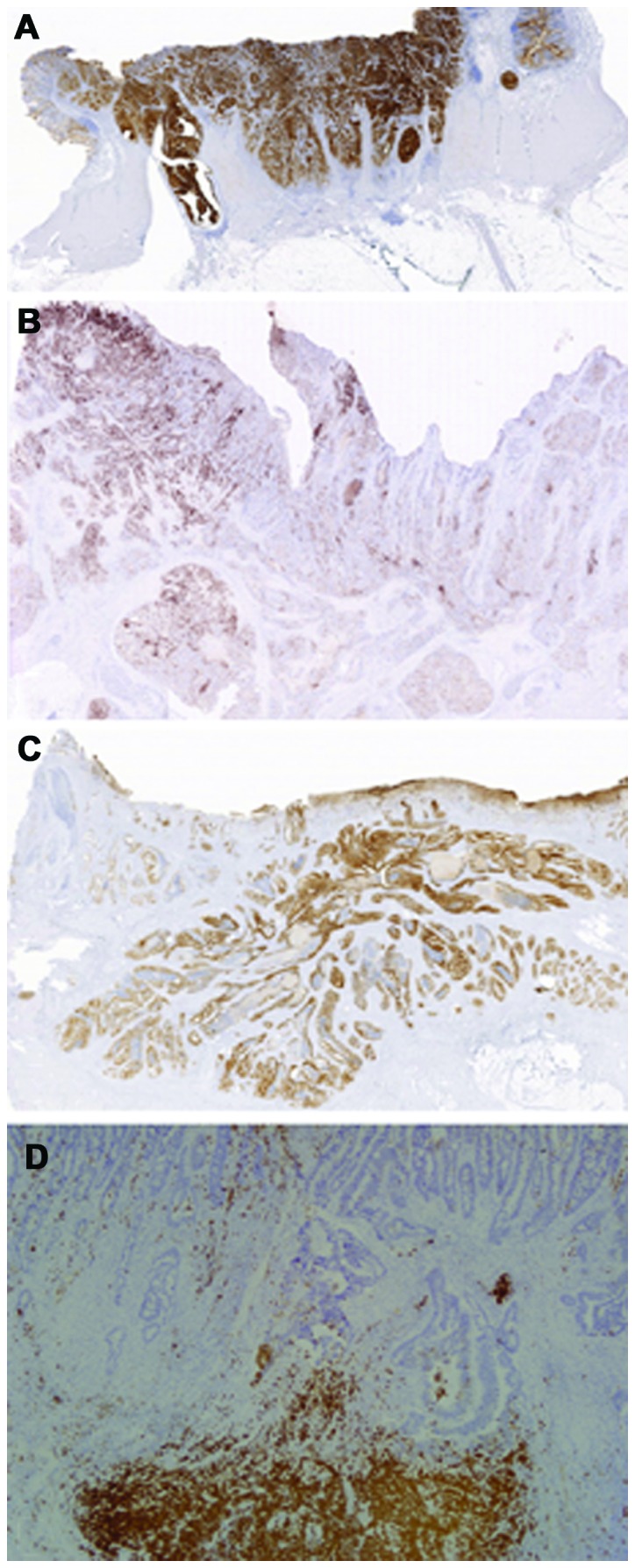
Representative S100P immunostaining in ulcerative tumor samples. (A) An ulcerative tumor showing diffuse, strong positivity (panoramic view). (B) Marked and (C) mild intratumoral heterogeneous immunostaining (panoramic view). (D) A tumor ruptured with abscess formation showing negative staining. Neutrophils scattering within the tumor and forming abscess displaying strong reactivity (magnification, ×200).

We then analyzed S100P protein expression in adenomas and paired samples of normal mucosa and adenocarcinomas using a rabbit monoclonal antibody. Immunohistochemically, only trophoblasts in the placenta showed strong a nuclear and weak cytoplasmic staining (Fig. 3A), which is identical to that of a previous study using a mouse monoclonal 18-9 antibody (24). The normal colon mucosa samples frequently showed absent or focal weak staining (Fig. 3B) as previously reported (24,25). Variable portions of dysplastic epithelial cells of low-grade adenomas displayed strong nuclear reactivity and weak reactivity (Fig. 3C), whereas dysplastic cells of high-grade adenoma showed more uniform and strong nuclear staining and moderate cytoplasmic staining (Fig. 3D). All 35 adenomas showed a moderate to strong S100P overexpression compared to the surrounding glands. Some glands had strong immunoreactivity concentrated in the apical and supranuclear regions without obvious nuclear staining (Fig. 3E). Apart from positivity in dysplastic epithelial cells, neutrophils displayed strong staining (Fig. 3F). Polypoid CRC frequently showed strong and uniform staining throughout the tumor (Fig. 4A and B). Notably, the polypoid part displayed much stronger staining (Fig. 4C) than that of the invasive front (Fig. 4D). Overall, 80% (24/30) and 20% (6/30) of polypoid CRC samples showed diffusely strong and moderate S100P protein overexpression, respectively. No tumors had weak or negative staining (Table III). By contrast, the ulcerative CRC samples displayed a wide range of expression levels; 15% (6/40) of the ulcerative CRC samples displayed strong and uniform staining as was noted in the polypoid CRC samples (Fig. 5A); 52.5% (21/40) had a moderate S100P overexpression in either marked (Fig. 5B) or mild (Fig. 5C) heterogenous patterns; 32.5% (13/40) showed weakly positive to negative staining (Fig. 5D). Overall, the overexpression of S100P protein was significantly greater in the polypoid CRC than that in the ulcerative CRC samples (P<0.001; Table III).

## Discussion

In the present study, we first identified S100P as the only upregulated gene preferentially associated with polypoid CRC by cDNA expression analyses. This finding was further substantiated by both qPCR and western blot analysis. S100P overexpression was observed early in the adenoma stage, and its immunoreactivity was predominantly localized in the nucleus and to a lesser extent in the cytoplasm. In the preliminary IHC analysis, 80% (24/30) of the polypoid CRC samples showed a strong S100P protein overexpression, whereas the ulcerative CRC samples showed a heterogeneous expression level with only 15% (6/40) displaying a strong overexpression. S100P protein overexpression was significantly associated with polypoid CRC (P<0.001). The level of promoter methylation by MSP appeared to be similar in these 2 types of CRC, indicating that DNA hypomethylation of the promoter may not be the key mechanism in the upregulation of S100P mRNA expression in polypoid CRC.

S100P, a 95-amino acid member of the S100 family of proteins containing 2 EF-hand calcium-binding motifs, was first isolated from the human placenta (17). The aberrant expression of S100P has been found in various types of cancer, including breast, prostate, pancreatic, lung and colon cancer, and its overexpression is putatively associated with drug resistance, metastasis and a poor clinical outcome (26–31). It has been demonstrated that S100P is overexpressed early in the preneoplastic adenoma stage (25,32). The early expression of S100P in adenoma indicates its important role in tumor initiation. A higher nuclear and cytoplasmic S100P expression in high-grade adenoma is compatible with its putative role in promoting cell proliferation and tumor progression. The cellular function of S100P in colorectal tumorigenesis has been investigated in *in vivo* and *in vitro* studies. Fuentes *et al* (33) demonstrated that S100P interacted with the receptor for advanced glycation end-products (RAGE) to stimulate SW480 colon cancer cell growth and migration and upregulate ErK phosphorylation and NF-κB activation *in vitro*. Jiang *et al* (34) knocked down S100P gene expression in DLD1 and SW620 colon cancer cells (high and low endogenous S100P expression, respectively) using lentivirus-mediated RNA interference, which resulted in the significant inhibition of cancer cell growth, migration and invasion *in vitro* and tumor growth and liver metastasis *in vivo*. The role of S100P in promoting cell growth and migration in the above-mentioned studies is compatible with the frequently strong overexpression in observed in polypoid CRC, since this tumor type characteristically shows florid upward outgrowth and expansion to obstruct the lumen. By contrast, the association of downregulated S100P expression with the inhibition of invasion is somewhat contradictory to the data of our IHC analysis. Our data showed that tumor cells of the invasive front in polypoid CRC showed much weaker staining than the non-invasive polypoid counterparts. Ulcerative CRC, characterized by the downward invasion into the bowel wall and peri-colonic soft tissue, displayed strong to weak S100P expression, indicating that S100P overexpression is not a key determinant in conferring the process of invasion. Although *in vitro* studies using cell lines are a powerful tool with several advantates, the results may vary according to the selection of cell lines. The clinicopathological significance of S100P expression in CRC requires further investigation using larger series of samples in well-designed studies.

Epigenetic mechanisms, particularly aberrant alterations in DNA methylation of several genes, play critical roles in the development of CRC (35). In pancreatic and prostate cancer, the hypomethylation in 5′CpG islands of the S100P gene promoter region has been shown to correlate significantly with S100P mRNA expression (22,36). Our data indicated that the methylation level of S100P did not differ significantly between polypoid and ulcerative CRC. Previous studies have demonstrated that gene-specific hypomethylation patterns may vary between different types of cancer (22). For example, a previous study demonstrated that the frequency of S100P hypomethylation did not differ significantly between pancreatic and breast cancer cell lines, whereas the frequency of hypomethylation of trefoil factor 2 (TFF2) and lipocalin 2 genes was significantly lower in the breast cancer cell lines than in pancreatic cancer cell lines (22). Therefore, it can be hypothesized that other regulatory factors other than S100P promoter hypomethylation contribute to the upregulation of S100P mRNA expression in CRC. Indeed, the study by Chandramouli *et al* demonstrated that prostaglandin E_2_/EP4 receptor signaling induced S100P expression in colon cancer cells through the ERK/MEK pathway (37). It has also been reported that S100P expression in prostate and breast cancer cells is controlled by androgen and progestin, respectively (38). Taking these data into consideration, it is likely that the regulation of S100P expression is a complex process influenced by the microenvironment and intrinsic nature of each organ and associated genetic abnormalities in tumorigenesis.

S100P can function as both an intracellular and extracellular signaling molecule (39). Calcium-dependent intracellular activities regulated by S100P include protein phosphorylation, cytoskeletal function and protection from oxidative cell damages (39). S100P has been shown to function extracellularly through its ability to activate RAGE (40). Our IHC analysis revealed that the subcellular localization of S100P was nuclear and/or cytoplasmic, similar to the data previously described for colon (24,25,33), prostate (28), pancreatic (29) and lung (30) cancer cells. We also observed intense S100P staining localized in the apical and supranuclear regions of the cytoplasm without nuclear staining, which is consistent with previous findings showing the apical location of calcium-selective ion channel (41). Additionally, intratumoral heterogeneous staining was particularly evident in ulcerative CRC. It is plausible that the subcellular localization of S100P reflects the functional status of the protein and its site of function. Therefore, distinct changes in the subcellular distribution of S100P are a reflection of its new activities. It is apparent that this dynamic process is controlled by complex regulatory mechanisms that have yet to be elucidated.

Although much progress in biomarker studies has been made over the past years, the majority of prognostic and predicative biomarkers are not used in clinical practice (42). Existing data collectively indicate that S100P is overexpressed in many types of cancer and that it may potentially serve as a diagnostic, prognostic and predictive marker and possibly also as a target of molecular therapy (38,39). Since S100P can be secreted extracellularly, it is conceivable that the basal serum levels of S100P should be determined before investigating its role as a reliable surrogate marker of CRC. It is particularly noteworthy that S100P expression is not restricted to neoplastic cells, but is also detectable in several normal cell types, including neutrophils. This fact must be taken into consideration when designing novel diagnostic and therapeutic applications based on S100P expression.

## Figures and Tables

**Table I:** The clinicopathological characteristics of the colorectal cancer patients included in the immunohistochemical analysis.

	Polypoid type	Ulcerative type
No. of patients	30	40
Gender		
Male	17	24
Female	13	16
Age (years)		
Average (range)	58 (30–79)	64 (36–78)
Tumor location		
Right-side colon	11	11
Left-side colon	4	16
Rectum	15	13
Tumor size (cm)		
Average (range)	4.1 (2.0–8.0)	4.7 (2.5–8.5)
Duke’s tumor stage		
A + B	19	20
C	11	16
D	0	4
Tumor differentiation		
Well	16	13
Moderate	14	23
Poor	0	4

**Table II:** Comparison of upregulated and downregulated genes between polypoid and ulcerative colorectal cancer.

Access no.	Gene name	Incidence	Polypoid fold change^a^ (mean + SD)	Incidence	Ulcerative fold change^a^ (mean + SD)	P-value
Upregulated genes						
Hs.2962	S100 calcium binding protein P	8	8.203±5.326	5	3.106±2.885	0.032
Hs.789	Chemokine (C-X-C motif) ligand 1 (melanoma growth stimulating activity, α)	7	5.212±4.858	4	2.309±1.620	0.131
Hs.423	Pancreatitis-associated protein	6	6.149±8.842	5	6.042±6.340	0.978
Hs.118787	Transforming growth factor, β-induced, 68 kDa	6	4.416±3.564	4	2.437±1.924	0.189
Hs.89690	GRO3 oncogene	6	5.094±4.650	3	1.492±2.077	0.065
Hs.458414	Hypothetical protein MGC27165	5	2.628±1.244	7	3.290±1.204	0.298
Hs.4158	Regenerating islet-derived lβ (pancreatic stone protein, pancreatic thread protein)	5	7.385±7.794	6	6.764±7.999	0.877
Hs.155596	BCL2/adenovirus E1B 19 kDa interacting protein 2	5	4.435±6.159	6	3.742±5.209	0.812
Hs.273321	Differentially expressed in hematopoietic lineages	5	5.795±6.185	6	4.964±6.661	0.8
Hs.129778	Serine protease inhibitor, Kazal type 4	5	4.194±5.219	4	4.770±6.989	0.93
Hs.105484	Regenerating gene type IV	4	2.061±2.532	4	1.820±1.765	0.629
Downregulated genes						
Hs.76325	Immunoglobulin J polypeptide, linker protein for immunoglobulin α and mu polypeptides	7	5.855	6	3.904	0.679
Hs.1650	Downregulated in adenoma	8	−10.268±6.787	8	−5.757±2.575	0.101
Hs.57975	Calsequestrin 2 (cardiac muscle)	5	−2.304±1.150	4	−1.835±1.478	0.491
Hs.381097	Metallothionein 1F (functional)	5	−2.864±2.134	6	−3.798±2.099	0.392
Hs.46847	TRAF and TNF receptor-associated protein	6	−2.914±1.039	4	−2.636±1.379	0.655
Hs.78224	Ribonuclease, RNase A family, 1 (pancreatic)	5	−2.626±2.151	4	−2.684±2.172	0.958
Hs.153261	Immunoglobulin heavy constant mu	5	−4.518±5.378	5	−6.765±10.794	0.606
Hs.248112	γ-aminobutyric acid (GABA) A receptor, α 4	5	−3.895±5.458	4	−3.681±6.296	0.943
Hs.366	Hypothetical protein MGC27165	7	−6.089±7.404	6	−5.718±3.426	0.751
Hs.433205	Metallothionein 1E (functional)	5	−3.004±2.137	6	−4.349±2.372	0.253
Hs.326248	Programmed cell death 4 (neoplastic transformation inhibitor)	7	−3.289±1.534	5	−2.593±2.049	0.455
Hs.84905	Cytokeratin 20	7	−4.440±3.257	4	−3.041±2.773	0.393
Hs.155048	Lutheran blood group (Auberger b antigen included)	3	−3.797±6.734	7	−4.312±2.989	0.548
Hs.87497	Butyrophilin, subfamily 3, member A2	5	−3.516±2.365	4	−2.770±2.654	0.562

a Fold change, corresponding to the ‘signal ratio’ of tumor/normal, was calculated from the ‘signal log ratio’.

**Table III:** Comparison of S100P expression levels between polypoid and ulcerative colorectal cancer.

	S100P expression score^a^				Overexpression^c^, n (%)	P-value
	0^b^	1–3	4–5	6–7		
Ulcerative (n=40)	0	13	21	6	6 (15)	<0.001
Polypoid (n=30)	0	0	6	24	24 (80)	

a Scoring was performed as described in Materials and methods.

b S100P protein expression level was defined as follows: 0, negative; 1–3, weak; 4–5, moderate; and 6–7, strong.

c Tumors with an expression score of 6–7 were defined as showing S100P overexpression.
